# An outpatient antibacterial stewardship intervention during the journey to JCI accreditation

**DOI:** 10.1186/2050-6511-15-8

**Published:** 2014-02-26

**Authors:** Ping Song, Wei Li, Quan Zhou

**Affiliations:** 1 Department of Pharmacy, the Second Affiliated Hospital, School of Medicine, Zhejiang University, Jiefang Road No. 88, Hangzhou, Zhejiang 310009, China; 2 Division of Medical Affairs, the Second Affiliated Hospital, School of Medicine, Zhejiang University, Hangzhou, Zhejiang 310009, Province, China

**Keywords:** Antibacterials, Continuous quality improvement, Drug utilization, Inappropriate prescribing, Outpatient health services, Pharmacoeconomics, Prescription auditing, Stewardship, Intervention

## Abstract

**Background:**

Antibacterial overuse, misuse and resistance have become a major global threat. The Joint Commission International (JCI) accreditation standards include quality improvement and patient safety, which is exemplified by antimicrobial stewardship. There are currently few reports on interventions to improve the quality of outpatient antibacterial prescribing.

**Methods:**

A before-after intervention study, aiming at antibacterial use in outpatients, was performed in a university-affiliated hospital with 2.8 million outpatient visits annually during the journey to JCI accreditation (March of 2012 - March of 2013). Comprehensive intervention measures included formulary adjustment, classification management, motivational, information technological, educational and organizational measures. A defined daily dose (DDD) methodology was applied. Pharmacoeconomic data and drug-related problems (DRPs) were statistically compared between the two phases.

**Results:**

The variety of antibacterials available in outpatient pharmacy decreased from 38 to 16. The proportion of antibacterial prescriptions significantly decreased (12.7% versus 9.9%, *P* < 0.01). The proportion of prescriptions containing the restricted antibacterials was 30.4% in the second phase, significantly lower than the value of 44.7% in the first phase (*P* < 0.01). The overall proportion of oral versus all antibacterial prescriptions increased (94.0% to 100%, *P* < 0.01) when measured as defined daily doses. Statistically significant increases in relative percentage of DDDs of oral antibacterials (i.e., DDDs of individual oral antibacterial divided by the sum of DDDs of all antibacterials) were observed with moxifloxacin, levofloxacin, cefuroxime axetil, ornidazole, clindamycin palmitate, cefaclor, amoxicillin and clarithromycin. Occurrence rate of DRPs decreased from 13.6% to 4.0% (*P* < 0.01), with a larger decrease seen in surgical clinics (surgical: 19.5% versus 5.6%; internal medicine: 8.4% versus 2.8%, *P* < 0.01). The total expenditure on antibacterials for outpatients decreased by 34.7% and the intervention program saved about 6 million Chinese Yuan Renminbi (CNY) annually.

**Conclusion:**

The one-year intervention program on outpatient antibacterial use during the journey to JCI accreditation reduced the expenditure on antibacterials, improved the appropriateness of antibacterial prescriptions. Quality improvements need integrated multifaceted intervention measures and long-term adherence to the antibiotic stewardship. Approach of i.v. to oral antibacterial switch, classification management, and motivational measures may play the most efficient role in changing antibacterial prescription practices.

## Background

Over use or the improper use of antibiotics can result in drug resistant bacteria as well as considerable expense to health care system [1]. According to a new report “*Antibiotic Resistance Threats in the United States, 2013”* issued by the U.S. Centers for Disease Control and Prevention (CDC), antibiotic resistance in the United States adds $20 billion in excess direct health care costs, with additional costs to society for lost productivity as high as $35 billion a year. The use of antibiotics is the single most important factor leading to antibiotic resistance. Up to 50 percent of all the antibiotics prescribed for people are not needed or are not prescribed appropriately [2].

It is imperative to create a culture of safety and quality within an organization that strives to continually improve the quality of antibiotic prescribing. A systematic review showed that antibiotic prescribing is a complex process influenced by factors affecting all the actors involved, including physicians, other healthcare providers, healthcare system, patients and the general public [3]. Sumpradit et al. reported that prescription behavior could be influenced by the factors like knowledge, attitudes, subjective norms, peer pressure, patient expectations, drug promotion, physician’s diagnostic skill and exposure to hospital formularies and standard therapeutic guidelines [4].

The benefits of antimicrobial stewardship have been well described and implemented in the inpatient setting [5-7]. Although studies of antimicrobial consumption in outpatient services have been conducted in countries like France, Jordan and the United States [8-10], there are very few intervention programs specially targeting the outpatient antibacterial use. A search of Medline between January 1 1993 and October 31 2013 revealed only two intervention studies when using the keywords: “antimicrobial stewardship and intervention and outpatient and prescribing”. One study evaluated the effect of clinician education coupled with audit and feedback on broad-spectrum antibacterial prescribing for pediatric outpatients with acute respiratory infections [11], and the other study assessed the effect of a computerized clinical decision support system (CDSS) on preventing misuse of fluoroquinolone and azithromycin for acute respiratory infections [12]. Compared with these interventions confined to special type of infection or particular class of antibiotics, multifaceted interventions for quality improvement in outpatient antibiotic prescribing has not been reported.

The fourth edition of Joint Commission International (JCI) accreditation standard defined irrational drug use as inappropriate drug, dose, frequency and route of administration, real or potential drug-drug interactions (DDIs), ignorance of allergy history, therapeutic duplications, and variation from organization criteria for use [13]. Rational antibacterial use is one of key measurable elements in quality improvement required by JCI. The Second Affiliated Hospital of Zhejiang University, School of Medicine, Zhejiang University, China (SAHZU) successfully passed the JCI accreditation as an academic medical center hospital on Feb 24 of 2013. Rational antibacterial use was listed as one of the nine patient safety goals in SAHZU in 2012–2013. A working group composed of infectious disease physicians, pharmacists, microbiologists, and administrators was established. This group sought to implement multifaceted interventions at the individual, organizational and policy levels to change antibacterial prescription practices [4]. During the journey to JCI accreditation, the SAHZU group performed an outpatient antibacterial stewardship intervention. The aim of this article was to discuss the effectiveness of such stewardship intervention in the outpatient setting and provide some reference for international counterparts.

## Methods

### Data collection

A before-after intervention study, focusing on antibacterial use in outpatient service was performed in SAHZU, a 2200-bed hospital with 2.8 million outpatient visits annually in Zhejiang Province, which has a population of approximately 54.4 million. The first phase was March of 2012 and the second phase was March of 2013. The study was approved by Ethics Committee at SAHZU and it was in compliance with the Helsinki Declaration.

Types of departments included in this study were as follows: (1) Surgical clinics: Burn, Cardiovascular Surgery, General Surgery Gynaecology, Neurosurgery, Oral and Maxillofacial Surgery, Orthodontics, Orthopaedics, Otolaryngology, Plastic Surgery, Prosthodontics, Surgical Oncology, Thoracic Surgery and Urology; (2) Internal medicine clinics: Allergy and Clinical Immunology, Cardiology, Dermatology, Endocrinology, Gastroenterology, Haematology, Infectious Diseases, Medical Oncology, Nephrology, Neurology, Oral Medicine, Respiratory Medicine and Rheumatology.

The total number of prescriptions for outpatients and total number of prescriptions containing antibacterials (ATC code J01) were derived from the prescription evaluation software embedded in the pharmacy management information system. Antibacterial expenditure and cost of all medications were calculated respectively. Herb medicine prescriptions, compulsorily prescribed separately from western medicines in China, were excluded when to calculate the total number of prescriptions for outpatients. A subset of 5% of the antibacterial-containing prescriptions was selected to evaluate for drug-related problems (DRPs) through random sampling, and was retrospectively evaluated by clinical pharmacists. Antibacterial-associated DRPs included inappropriate drug combination, dose, dosing frequency and administration route, use beyond approved indications, discordance between diagnosis and purpose of medication use, mismatches between antibacterial spectrum and the patient’s infection, abuse of intravenous (i.v.) medications instead of oral alternatives, ignorance of patient’s concomitant diseased conditions and other miscellaneous problems.

Data on outpatient antibacterial use were collected using the Anatomical Therapeutic Chemical (ATC)/defined daily dose (DDD) method (WHO, version 2013) [14,15]. Number of defined daily doses, also called DDDs, was calculated as total dose consumed divided by DDD. Relative percentage of DDDs of each oral antibacterial was calculated as DDDs of individual oral antibacterial divided by the sum of DDDs of all antibacterials. Daily expenditure is calculated as overall expenditure divided by DDDs. Occurrence rate of DRPs was calculated as the number of DRPs divided by the number of randomly selected antibacterial prescriptions.

The data presented in the study is available in the archives of Drug & Therapeutics Committee (DTC) of SAHZU. Access and use of these data need permission from the SAHZU DTC.

### Comprehensive intervention measures

#### Formulary adjustment & classification management

Antibacterials were classified as non-restricted (also called “first line”), restricted (“second line”), or special-grade (“third line”). Non-restricted antibacterials refer to those with relatively low price, proven safety and clinical efficacy and little effect on bacterial resistance. Restricted antibacterials refer to those with proven safety and clinical efficacy, but relatively high price and greater impact on bacterial resistance. Special-grade antibacterials are those with common or serious adverse reactions, tendency of producing rapid bacterial resistance or inadequate clinical efficacy and safety data. Each grade of antibacterial matched corresponding prescribing privileges for physicians. The antibacterial formulary was updated (Table 1). i.v. antibacterials were deleted from the formulary of outpatient pharmacy. i.v. to oral antibacterial switch therapy was encouraged in outpatient services according to the principle of antimicrobial pharmacology (Table 2).

**Table 1:** Formulary adjustment & classification management

**First phase (March, 2012)**			**Second phase (March, 2013)**		
**Category**	**Antibacterials**	**Level**	**Category**	**Antibacterials**	**Level**
Macrolides	Oral clarithromycin, Oral azithromycin, Oral erythromycin, i.v. erythromycin	1	Macrolides	Oral clarithromycin, Oral azithromycin, Oral roxithromycin	1
	i.v. azithromycin	2	Cephalosporins		
Cephalosporins			The first generation	Oral cephradine	1
The first generation	Oral cephradine	1	The second generation	Oral cefuroxime axetil, Oral cefaclor	1
	i.v. cefathiamidine	2		Oral cefprozi	2
The second generation	Oral cefuroxime axetil, Oral cefaclor, i.v. cefuroxime sodium	1	The third generation	Oral cefdinir	2
	Oral cefprozi, i.v. cefotiam	2	Fluoroquinolones	Oral levofloxacin	1
The third generation	Oral cefetamet pivoxil hydrochloride, Oral cefdinir, i.v. ceftizoxime	2		Oral moxifloxacin	2
	i.v. ceftriaxone sodium	1	Penicillins	Oral amoxicillin	1
Cephamycins	i.v. cefmetazole sodium, i.v. cefminox sodium	2	Beta-lactam/beta-lactamase inhibitor combinations	Oral amoxicillin and clavulanate potassium	1
Monocyclic beta-lactam	i.v. aztreonam	3	Nitroimidazoles	Oral ornidazole	2
Fluoroquinolones	Oral levofloxacin, i.v. levofloxacin, i.v. ciprofloxacin	1	Tetracyclines	Oral minocycline	1
	Oral moxifloxacin, i.v. moxifloxacin	2	Lincosamides	Oral clindamycin palmitate	1
Aminoglycosides	i.v. etimicin	2	Miscellaneous	Oral sulfamethoxazole/trimethoprim	1
	i.v. streptomycin sulfate	1			
Penicillins	i.v. benzylpenicillin sodium, Oral amoxicillin	1			
	i.v. sulbenicillin sodium	2			
Beta-lactam/beta-lactamase inhibitor combinations	Oral amoxicillin and clavulanate potassium	1			
	i.v. piperacillin/sulbactam, i.v. cefoperazone/sulbactam	2			
Nitroimidazoles	Oral ornidazole, i.v. ornidazole	2			
Tetracyclines	Oral minocycline	1			
Lincosamides	Oral clindamycin palmitate, i.v. clindamycin	1			
Miscellaneous	i.v. fosfomycin sodium	1			

Notes: Level 1: non-restricted (also called “first line”) antibacterials; Level 2: restricted (“second line”) antibacterials; Level 3: special-grade (“third line”) antibacterials.

**Table 2:** Approach of i.v. to oral antibacterial switch therapy

**i.v. antibacterials**	**Oral antibacterials**	**Daily expenditure ratio (oral to i.v.)**
Fluoroquinolones		
i.v. moxifloxacin	Moxifloxacin tablets	0.10
i.v. levofloxacin	Levofloxacin tablets	0.13
Cephalosporins		
The first generation		
i.v. cefathiamidine	Cefradine capsules	0.056
	The second generation cephalosporins and macrolides	0.041-0.14 (median: 0.078)
The second generation		
i.v. cefotiam	Cefaclor capsules	0.092
i.v. cefuroxime sodium	Cefaclor SR tablets	0.99
	Cefprozi tablets	0.15
	Cefuroxime axetil tablets	0.061
The third generation		
i.v. ceftizoxime sodium	Cefdinir capsules	0.35
i.v. ceftriaxone sodium	Cefetamet pivoxil tablets	0.042
Macrolides		
i.v. azithromycin	Azithromycin tablets	0.29
	Roxithromycin tablets	0.13
	Clarithromycin SR tablets	0.088
	Clarithromycin tablets	0.21
Aminoglycosides		
i.v. etimicin	Levofloxacin tablets	0.097
	Cefdinir capsules	0.34
	Cefetamet pivoxil tablets	0.090
Nitroimidazoles		
i.v. ornidazole	Ornidazole tablets	0.017
Lincosamide		
i.v. clindamycin	Clindamycin palmitate tablets	1.11

Daily expenditure was expressed in Chinese Yuan Renminbi (CNY); i.v.: intravenous; SR: sustained release. Daily expenditure ratio (oral to i.v.) was calculated as daily expenditure of oral antibacterial divided by daily expenditure of the corresponding i.v. antibacterial.

#### Motivational interventions

The director of each clinical department was asked to sign a goal-setting responsibility plan for antibacterial use with each director of clinical department. Reports of prescription-related near misses were encouraged through a voluntary online reporting system. Retrospective appropriateness evaluation of antibacterial-containing prescriptions was performed monthly by clinical pharmacists and the results were discussed in the meeting of the DTC and published on the hospital local area network. “Dear doctor” letters were sent from the DTC to physicians. Physicians were given the opportunity to present evidence and argument against the results of audit-feedback during a seven-day public notice period. Physicians who wrote inappropriate prescriptions would generally face a fine according to the severity of the DRPs. The level of fine was divided into three grades [low-grade: 100 Chinese Yuan Renminbi (CNY); medium-grade: 200 CNY; high-grade: 300 CNY]. Prescribing privilege of the physician would be revoked if there was a second instance of a high-grade error.

#### Information technological interventions

Web-based prescription screening software and a CDSS for antibacterial prescribing, embedded in electronic medical records (EMRs), were implemented. Drug information resources were updated, specifying maximum dose, contraindications and special precautions. An improved interface was created between the pharmacy management information system for prescription auditing which displayed both medication information (i.e., medication name, dose, administration route, dose frequency and current medications) and patient’s key information (e.g., patient name, identification number, age, diagnosis, allergy history, body weight, body surface area, nutrition status and clinical laboratory test results such as hepatic and renal function, blood routine examination, and serum drug levels).

#### Educational interventions

Physicians were instructed to follow the clinical guidelines. They also must record any real or potential allergies or sensitivities in the electronic medical record for outpatients and note the results of allergy skin tests when prescribing special medications for outpatients. Lectures were given, providing key opportunities for physicians to learn about the topics of medication management therapy, DDIs, medication errors, adverse drug reactions, therapeutic monitoring and typical cases of irrational physician orders. Physicians and pharmacists should receive specific training in antibacterial prescription before they are granted different levels of prescribing or dispensing privileges.

### Outcome measures

The outcome measures included proportion of prescriptions containing antibacterials, proportion of prescriptions containing non-restricted antibacterials, proportion of prescriptions containing restricted antibacterials, proportion of prescriptions containing special-grade antibacterials, total expenditure on antibacterials for outpatients, proportion of expenditure on antibacterials relative to all medications, proportion of expenditure on i.v. antibacterials relative to all antibacterials, sum of DDDs of antibacterials, proportion of DDDs of all oral antibacterials relative to all antibacterials, relative percentage of DDDs of each oral antibacterial, number of DRPs, occurrence rate of DRPs, occurrence rate of DRPs made by surgeons, occurrence rate of DRPs made by internal medicine physicians, occurrence rate of each DRP subtype and number of physicians who received fines during the study period.

### Statistical analysis

A descriptive analysis was performed. Pearson’s Chi-square test was used for testing percentage differences between two groups. Student’s *t*-test was used for statistical comparisons between the means of two groups. A *P* value < 0.05 was considered to be statistically significant. A *P* value < 0.01 was considered to be highly significant.

## Results

General information and pharmacoeconomic data on antibacterial prescriptions are presented in Table 3. During the first phase, average daily expenditure on i.v. antibacterials was approximately 12 times that of oral antibacterials [193.5 ± 172.2 CNY versus 16.1 ± 13.1 CNY, *P* < 0.01]. The changes in relative percentage of DDDs of oral antibacterials between the two phases are listed in Table 4. Statistically significant increases in relative percentage of DDDs of oral antibacterials were observed with moxifloxacin, levofloxacin, cefuroxime axetil, ornidazole, clindamycin palmitate, cefaclor, amoxicillin and clarithromycin. Cefaclor demonstrated the largest increase in utilization. Cefuroxime axetil was the most usually prescribed antibacterials during each phase. The total expenditure for outpatient antibacterials decreased by 34.7% and the intervention program saved 0.51 million CNY per month. It is estimated to save about 6 million CNY annually.

**Table 3:** General information and pharmacoeconomic data on antibacterial prescriptions

**Indicators**	**First phase**	**Second phase**
	**(March, 2012)**	**(March, 2013)**
Kinds of antibacterials in outpatient pharmacy	38	16
Kinds of the third line antibacterials	1	0
Kinds of the second line antibacterials	17	4
Kinds of the first line antibacterials	20	12
Kinds of i.v. antibacterials	22	0
Kinds of oral antibacterials	16	16
Total number of prescriptions for outpatients	88425	90459
Total number of prescriptions containing antibacterials	11194	8920
Proportion of prescriptions containing antibacterials^#^	12.7%	9.9%
Proportion of prescriptions containing non-restricted antibacterials^#^	55.1%	69.6%
Proportion of prescriptions containing restricted antibacterials^#^	44.7%	30.4%
Proportion of prescriptions containing special-grade antibacterials^#^	0.14%	0
Total expenditure on antibacterials for outpatients (million CNY)	1.4760	0.9644
Proportion of expenditure on antibacterials relative to all medications	6.9%	4.1%
Proportion of expenditure on i.v. antibacterials relative to all antibacterials	30.5%	0
Sum of DDDs of antibacterials for outpatients	65930	61403
Proportion of DDDs of oral antibacterials for outpatients relative to all antibacterials^#^	94.0%	100%

Notes: ^#^*P* < 0.01 (first phase *vs* second phase). Differences between the two phases were tested for statistical significance using Pearson’s Chi-square test. A *P* value < 0.05 was considered to be statistically significant. A *P* value < 0.01 was considered to be highly significant. CNY: Chinese Yuan Renminbi. DDDs: number of defined daily doses (total dose consumed/defined daily dose). Daily expenditure = overall expenditure/DDDs.

**Table 4:** Comparison in relative percentage of DDDs of oral antibacterials before and after intervention

**Oral antibacterials**	**Relative percentage of DDDs**	
	**First phase (March, 2012)**	**Second phase (March, 2013)**
Cefuroxime axetil tablets^#^	10.6%	13.2%
Levofloxacin tablets^#^	9.6%	12.6%
Moxifloxacin tablets^#^	8.6%	9.1%
Amoxicillin^#^	8.1%	11.4%
Cefdinir capsules	7.0%	7.2%
Clarithromycin tablets^#^	5.7%	7.4%
Cefradine capsules^#^	3.1%	1.8%
Ornidazole tablets^#^	2.3%	2.8%
Cefaclor SR tablets & Cefaclor capsules^#^	1.8%	9.3%
Cefprozi tablets^#^	1.72%	1.2%
Clindamycin palmitate tablets^#^	0.48%	1.2%

Notes: ^#^*P* < 0.01 (first phase *vs* second phase). Differences between the two phases were tested for statistical significance using Pearson’s Chi-square test. A *P* value < 0.01 was considered to be highly significant. DDDs: number of of defined daily doses (total dose consumed/defined daily dose). Relative percentage of DDDs: DDDs of individual antibacterial divided by the sum of DDDs of all antibacterials. SR: sustained release.

DRPs derived from randomly selected antibacterial prescriptions for outpatients are listed in Table 5. The proportion of injectable antibacterials prescribed dramatically decreased from 15.6% in the first phase to 0.2% in the second phase (*P* < 0.01). The occurrence rate of DRPs decreased from 13.6% in the first phase to 4.0% in the second phase (*P* < 0.01). In the first phase, surgeons demonstrated a significantly higher DRP rate than internal medicine physicians (19.5% versus 8.4%, *P* < 0.01); however, this difference was not seen in the second phase. Overall rates of DRPs decreased for both internal medicine physicians and surgeons when comparing the first and second phases (surgeons: 19.5% versus 5.6%; internal medicine physicians: 8.4% versus 2.8%, *P* < 0.01). The magnitude of decrease in occurrence of DRPs was more profound in surgeons. Significant differences between two phases were also observed with inappropriate co-medication with other antibacterials, mismatches between antibacterial spectrum and the patient’s infection, and abuse of i.v. medications instead of oral alternatives (*P* < 0.01).

**Table 5:** Drug-related problems derived from randomly selected antibacterial prescriptions for outpatients

**Indicators**	**First phase**	**Second phase**
	**(March, 2012)**	**(March, 2013)**
Number of randomly selected antibacterial prescriptions for outpatients^*^	559	446
Number of DRPs	76	18
Occurrence rate of DRPS^#^	13.6%	4.0%
Occurrence rate of DRPs made by surgeons^∆#^	19.5%	5.6%
Occurrence rate of DRPs made by internal medicine physicians^#^	8.4%	2.8%
Occurrence rate of each subtype of DRP		
(1) Inappropriate coadministration with non-antibacterials	4 (0.7%)	6 (1.3%)
(2) Inappropriate co-medication with other antibacterials^#^	17 (3.0%)	3 (0.7%)
(3) Inappropriate dosing frequency	8 (1.4%)	3 (0.7%)
(4) Inappropriate dose	1 (0.2%)	2 (0.4%)
(5) Inappropriate administration route	1 (0.2%)	0
(6) Use beyond approved indications	1 (0.2%)	2 (0.4%)
(7) Discordance between diagnosis and purpose of medication use	8 (1.4%)	2 (0.4%)
(8) Mismatches between antibacterial spectrum and the patient’s infection^#^	15 (2.7%)	0
(9) Abuse of i.v. medications instead of oral alternatives^#^	16 (2.9%)	0
(10) No diluent for i.v. antibacterials	1 (0.2%)	0
(11) Ignorance of patient’s other diseases	4 (0.7%)	0

Notes: ^#^*P* < 0.01 (first phase *vs* second phase). ^∆^*P* < 0.01 (surgeons *vs* internal medicine physicians). Differences between two groups were tested for statistical significance using Pearson’s Chi-square test. A *P* value < 0.05 was considered to be statistically significant. A *P* value < 0.01 was considered to be highly significant. DRPs: drug-related problems.

During the study period, sixty-one physicians were fined due to inappropriate prescribing. One surgeon received a fine of 13000 CNY at a monthly DTC meeting following audit-feedback. The inappropriateness of antibiotics prescribed by this surgeon was reflected in improper combination antibiotic treatment (19 prescriptions), lack of clear clinical features of infection plus improper combination antibiotic treatment (26 prescriptions), off-label use (2 prescriptions), inappropriate antibiotic choice (1 prescription), and inappropriate antibiotic choice plus improper combination antibiotic treatment (3 prescriptions). For example, he prescribed isepamicin sulfate-levofloxacin combination for a patient with enlarged axillary lymph nodes in the right axillary region, ceftizoxime-levofloxacin combination for treatment of lower extremity infection, and cefotiam-isepamicin sulfate-ornidazole combination for patient with acute appendicitis.

## Discussion

The significant decrease in the proportion of antibacterial prescriptions was an interesting outcome, as outpatient turnover and total number of prescriptions for outpatients did not decrease between the two phases. The increased proportion of prescriptions containing non-restricted antibacterials and the decreased proportion of prescriptions containing restricted antibacterials indicated that the stewardship efforts were successful.

The i.v.-to-oral switch therapy may reduce length of hospital stay, healthcare costs and risk of complications related to i.v. access [16,17]. For outpatients, oral moxifloxacin is a superior choice versus the i.v. formulation, with the advantages including one-tenth of the daily expenditure of i.v. medication, convenient administration schedule (just one tablet ingestion once daily versus 90-min intravenous drip), lower occurrence rate of infusion-related side effects (e.g., thrombophlebitis), and omission of additional non-drug cost associated with infusion therapy (e.g., clinical work load, hospital waste and administration cost such as needles, syringes, dressings, antiseptics and administration set). Oral levofloxacin and ornidazole have near 100 percent bioavailability; therefore a comparable exposure to the i.v. regimen may be achieved after oral administration [18,19]. i.v. to oral switch programme for clindamycin and cefuroxime have been also proved with great economic advantages [20,21]. Clinicians should consider oral antibacterials as soon as possible for outpatients who need infection control. The DDDs value represents the tendency of drug use, with higher DDDs indicating more frequent utilization [22]. Sum of DDDs of antibacterials for outpatients in the second phase was lower than that in the first phase (61403 versus 65930). Moreover, in this program, statistically significant changes of proportion of DDDs of eight oral antibacterials between two phases indicated that i.v. to oral switch therapy approach was basically successful. A decrease in the relative percentage of DDDs for cefprozi tablets may have been due to the introduction of cefaclor sustained release formulation into SAHZU in July, 2012. A decrease in the relative percentage of DDDs for cefradine capsules may have been in part due to increased awareness of its potential renal toxicity (e.g. hematuresis) among physicians and pharmacists.

Occurrence rates of DRPs between surgical clinics and internal medicine clinics exhibited significant difference in the first phase, but not in the second phase, indicating that surgeons were previously less likely to appropriately prescribe outpatient antimicrobials than internal medicine physicians; however, upon intervention, surgeon prescribing practices improved greatly. A systematic review revealed that physicians’ attitudes were the most influential intrinsic factor influencing antibacterial prescribing and healthcare system-related factors like time pressure and corresponding policies/guidelines implemented were the most common extrinsic factors [23]. A substantial number of surgeons were found to have suboptimal knowledge about antibacterial use and prescribing habits in the first phase. Besides of continuing education and training directed to them, fines were imposed on physicians who refused to mend their ways after repeated education. Furthermore, the outpatient service environment and treatment process were further improved. Such strenuous efforts improved the quality of antibacterial prescribing.

DRPs associated with “use beyond approved indications” were identified. Moxifloxacin tablet was prescribed for treatment of urinary tract infection in a prescription. However, package insert of moxifloxacin tablet specifies that the formulation is only indicated for the treatment of adults (≥ 18 years of age) with infections caused by susceptible isolates of the designated microorganisms in the conditions listed below: acute bacterial sinusitis, acute bacterial exacerbation of chronic bronchitis, community acquired pneumonia, uncomplicated skin and skin structure infections and complicated intra-abdominal infections. Not all fluoroquinolones can be used for urinary tract infections based on their pharmacokinetic profiles [24]. Treatment of infections in the urine is a common misuse of moxifloxacin, and clinicians should be careful about use of this agent for these infections since moxifloxacin achieves considerably lower concentrations in the urine than other fluoroquinolones and it is not approved for this indication. This study showed that the use of 0.4% of antibacterial prescriptions in the second phase was still beyond approved indications. Although off-label manner of prescribing cannot always be avoided, physicians should only use unapproved drugs in cases when suitable alternatives are unavailable and there are scientific evidence regarding safety and effectiveness [25,26].

The incidence of DRPs such as bug-drug mismatches and abuse of i.v. medications rather than highly bioavailable oral alternatives were successfully reduced in the second phase (*P*<0.01). In the first phase, sulbenicillin, a penicillin antibiotic active against *P. aeruginosa*, was severely misused for treatment of infections which were not commonly caused by *P. aeruginosa.* For example, sulbenicillin was prescribed for outpatients who got tooth extraction. Finally, the DTC eliminated sulbenicillin from the antibiotic formulary for outpatient clinical practice. Special trainings sessions were directed toward dentists to prevent the overuse and misuse of antibacterials, which was successful.

With regard to the indicator “Inappropriate coadministration with non-antibacterials”, no obvious improvement was observed. In China, antibacterials could be prescribed with other western medicines in the same prescription sheet. Coadministration of antibacterials and probiotics were observed in both phases. Spaced dosing was not specified when physicians wrote prescriptions. Since probiotics contain live microorganisms, concurrent administration of antibacterials could kill a large number of the organisms, reducing the efficacy of the *Lactobacillus* and *Bifidobacterium* species. However, all of patients in the two phases were instructed by pharmacists to separate administration of antibacterials from these bacteria-derived probiotics by at least two hours.

Although a significant reduction in occurrence of DRPs was observed after the intervention program, there still were 18 DRPs in the second phase. It indicated of further opportunities for improvement. In theory, the appropriateness of all prescriptions should be audited by pharmacists. In SAHZU, prospective prescription audit is a routine pharmaceutical service for inpatients. However, outpatient pharmacy has not yet established such working process due to big workload (5000 prescriptions each day). This hasn’t been possible, due to the relatively insufficient personnel and the particularity of outpatient service medical treatment in comparison with inpatient service (about 10000 outpatients each day versus 2200 inpatients each day). In Mainland of China, there is hardly any hospital which practices prospective prescription audit promptly in outpatient service when physicians prescribe via electronic prescribing system. Some large hospitals in China take the following measures: (1) Install online prescription screening software embedded in electronic prescribing system and pharmacy administration system, allowing severe DRPs to be automatically intercepted when physicians are prescribing. (2) Pharmacists will contact the corresponding physicians if they detect DRPs when outpatients hand over the prescriptions to pharmacists. Such interventions bring potential problems, and are likely to cause the tension between the hospital and patients (i.e., a patient would be reluctant to go back to the physician’s clinic for prescription revision because it will be perceived as a waste of time, and the physician’s professional image might be damaged).

Suboptimal prescribing habits involving antibiotics in China is partly associated with economic incentives, driven by non-standardized drug-promotions by pharmaceutical companies. However, many medical disputes between patients and hospitals are associated with irrational drug use. Therefore, education is an essential element of an intervention program designed to influence prescribing behavior and can provide a knowledge basis that will enhance the acceptance of stewardship strategies. Communication is also important among physicians, pharmacists and administrators before initiating intervention programs. Consideration should be given to all staff involved (i.e., values, personality, perceptions, emotions and ability). The working mode of the DTC on outpatient antibacterial stewardship intervention, especially allowing physicians to present evidence and argument to the results of audit-feedback during a seven-day public notice period, was pivotal to the willing acceptance of punishment by physicians who made severe DRPs. Rational antibiotic use, to a certain extent, is a health administration issue more so than a professional issue.

A literature review of clinical and economic outcomes of pharmaceutical services related to antibacterial use showed that the most frequently observed outcomes with a positive impact were appropriateness of prescribing and cost savings [27]. Although the study in SAHZU showed some positive outcomes, it had some limitations which were shown as follows: (1) Follow-up of outpatients’ therapeutic outcome was not conducted; (2) Patient adherence to treatment regimens were not monitored so that prescribing data may not accurately represent actual antibacterial consumption); (3) Multiple comparisons for the same dataset might increase type I error in showing the impact of interventions, and for these kind of studies interrupted time-series analysis would be more reliable method [28].

## Conclusion

The effects of an outpatient antibacterial stewardship intervention were examined in an academic medical center hospital during the journey to JCI accreditation. The intervention program reduced the expenditure on antibacterials, improved the appropriateness of antibacterial prescriptions. Quality improvements need continuous efforts, integrated multifaceted intervention measures and long-term adherence to the antibiotic stewardship. Approach of i.v. to oral antibacterial switch therapy, classification management, and motivational measures may play the most direct and efficient role in changing antibacterial prescription practices.

## Competing interests

The authors declare that they have no competing interests.

## Authors’ contributions

SP, LW and ZQ conceived and designed research; ZQ collected data and performed data analysis; and SP and ZQ wrote the paper. All authors’ read and approved the final manuscript.

## Pre-publication history

The pre-publication history for this paper can be accessed here:

http://www.biomedcentral.com/2050-6511/15/8/prepub
